# Cumulative life events, traumatic experiences, and psychiatric symptomatology in transition-aged youth with autism spectrum disorder

**DOI:** 10.1186/s11689-016-9160-y

**Published:** 2016-07-27

**Authors:** Julie Lounds Taylor, Katherine O. Gotham

**Affiliations:** 1 Department of Pediatrics and Vanderbilt Kennedy Center, Vanderbilt University School of Medicine, PMB 40 – 230 Appleton Pl., Nashville, TN 37203 USA; 2 Department of Psychiatry and Behavioral Sciences, Vanderbilt University School of Medicine, Vanderbilt Kennedy Center, 1200 21st Ave. S, suite 2272, Nashville, TN 37203 USA

**Keywords:** Autism spectrum disorder, Trauma, Life events, Depression, Anxiety, Internalizing

## Abstract

**Background:**

Co-occurring mood and anxiety symptomatology is commonly observed among youth with autism spectrum disorders (ASD) during adolescence and adulthood. Yet, little is known about the factors that might predispose youth with ASD to mood and anxiety problems. In this study, we focus on the role of cumulative stressful life events and trauma in co-occurring psychopathology among youth with ASD who are preparing to exit high school. Specifically, we examined the distribution of cumulative life events and traumatic experiences and their relations with mood and anxiety symptomatology.

**Methods:**

Participants included 36 youth with ASD, all of whom were in their last year of high school. Cumulative life events and trauma were assessed by parent report. Mood and anxiety symptomatology was determined using a variety of methods (structured interview, questionnaire, self- and informant report). Frequencies were used to examine the distributions of cumulative life events (count of total events) and trauma (coded into any trauma vs. no trauma), as well as mood and anxiety symptomatology (categorized into clinical-level, sub-threshold, or none for each). Bivariate relations between life events/trauma and mood/anxiety symptomatology were assessed using analysis of variance and chi-square. Ordinal logistic regression models were used to test whether significant bivariate relations remained after controlling for the sex of the youth with ASD and his/her IQ.

**Results:**

Over 50 % of youth had experienced at least one trauma. Nearly one half had clinical-level mood or anxiety symptomatology. There was a statistically significant relation between absence/presence of trauma and mood symptomatology; nearly 90 % of the youth with clinical-level mood symptoms had at least one trauma, compared to 40 % of those with no mood symptomatology.

**Conclusions:**

Our findings suggest that contextual factors such as trauma might be important for the development of mood symptomatology in individuals with ASD. Although this idea is well-accepted in typically developing populations, contextual factors are rarely studied in investigations of psychopathology or transition outcomes in ASD. Given the high rates of psychiatric comorbidities in this population, future research should continue to identify the range of possible factors—both behavioral and contextual—that might influence the emergence of these disorders.

**Electronic supplementary material:**

The online version of this article (doi:10.1186/s11689-016-9160-y) contains supplementary material, which is available to authorized users.

## Background

High rates of psychiatric comorbidities are common among individuals with autism spectrum disorders (ASD) [1, 2]. Not only is co-occurring symptomatology impairing in its own right, but it places significant limitations on these youth’s ability to successfully transition out of high school and into the adult world [3, 4]. Yet little is known about the factors related to the emergence of these disorders. The present study focuses on two related factors that consistently predict psychopathology in many other samples but are understudied in ASD: cumulative stressful life events and traumatic experiences.

Mood and anxiety disorders are the most widespread psychiatric comorbidities in adolescents and adults with ASD. Studies consistently find elevated rates of anxiety disorders and symptomatology in these individuals (for reviews, see [5, 6]). Studies that use self-reports of current depressive symptoms tend to find clinical-level symptomatology in a large minority of adult ASD samples [7, 8]. Using the more rigorous Structured Clinical Interview for DSM-IV Disorders, 50–70 % of adults with ASD are reported to have a lifetime diagnosis of mood disorder, and 50 % have lifetime anxiety disorders [9, 10].

To date, much of the research on correlates of internalizing disorders in individuals with ASD has focused on characteristics of the person. For example, both anxiety and depression have been linked to older chronological age, higher IQ, and greater insistence on sameness [1, 11–13]. In addition to individual characteristics, influential theories of human development suggest that contextual factors also play a critical role in the emergence of psychopathology [14, 15]. To date, the main contextual factor that has been studied in relation to psychopathology in ASD is socioeconomic status, with studies finding mixed results [16]. Clearly more research is needed to understand the range of factors that contribute to the high rates of psychopathology observed among individuals with ASD.

One contextual factor that might be related to the emergence of mood or anxiety problems among these adolescents and adults is trauma. Traumatic events are defined in the DSM-5 as “direct personal experience of an event that involves actual or threatened death or serious injury, or other threat to one’s physical integrity; or witnessing an event that involves death, injury, or a threat to the physical integrity of another person; or learning about unexpected or violent death, serious harm, or threat of death or injury experienced by a family member or other close associate.” This is often operationalized as events such as abuse (physical, sexual), natural disasters, witnessing of violence, or terrorism [17]. There is a well-established link from trauma to anxiety (especially post-traumatic stress disorder) and mood disorders in the general population (e.g., [18–21]). This association likely results from the overwhelming biological and psychological stress response to trauma leading to poor emotion regulation and diminished ability to cope with subsequent stressors, even if these are not traumatic in themselves (see [22] for an overview). Modulation of the stress response by the limbic-hypothalamic-pituitary-adrenal (LHPA) axis is likely involved in this cascade effect linking trauma and negative mental health outcomes.

Although they go by many names (e.g., adverse childhood experiences, major/significant life events, traumatic events), the accumulation of major and potentially traumatic life events is also consistently related to psychopathology in the general population [23, 24]—particularly to depression. This class of events includes the aforementioned “traumas” but also encompasses a broader set of experiences that are highly likely to be experienced as stressful such as (for example) parental divorce, a family member who goes to prison, or household substance abuse (for reference, see the Adverse Childhood Experiences study [25] and Turner et al. [18]). Data from many large-scale studies, including those of typically developing adults, international adoptees, and economically disadvantaged youth, have found strong relationships between the number of these stressful life events experienced throughout childhood and later anxiety and mood disorders [26–28].

Stressful life events and trauma have also been linked to comorbid mood and anxiety problems in adults with an intellectual disability (ID), both in terms of specific events such as sexual abuse or death of a parent, as well as using general cumulative indices of stressful events (for a review, see [29]). Although most of those studies focused on the impact of recent life events on comorbid psychopathology, Martorell et al. [30] examined the individual contributions of the sum of *lifetime* traumas as separate from a cumulative index of *recent* stressful life events. Their results suggested that, when examined simultaneously, lifetime trauma was more closely linked to comorbid disorders among adults with ID than were recent stressful events.

Although consistently associated with psychiatric disorders in typically developing populations and in those with ID, few studies have examined the role of stressful life events and trauma in the development of psychopathology among individuals with ASD [22], with only one that has examined the impact of cumulative lifetime events. Specifically, Ghazuiddin and colleagues [31] found that 82 % of children with ASD and depression had experienced at least one stressful life event over their lifetime (such as a change in group home, bereavement, or family sickness), compared to 45 % of children with ASD without depression. Milovanov and colleagues [32] found that for adults with Asperger syndrome, experiencing more stressful life events in the past 2 months (e.g., change in roommates, financial problems, recent trauma/abuse) was related to greater distress. Other studies have examined the psychiatric implications of specific traumas for individuals with ASD—such as abuse and/or neglect [33, 34] or natural disasters [35]—and the types of traumas related to the emergence of post-traumatic stress disorder [36]. More work is needed to understand the role that cumulative stressful life events and traumatic experiences might play in the emergence of psychiatric comorbidities in ASD.

### The present study

In the present study, we used a small but well-characterized sample of transition-aged youth with ASD to examine the relations between cumulative stressful life events, trauma, and co-occurring mood and anxiety symptomatology. This study extends the extant research in a number of important ways. Instead of focusing on the psychiatric sequelae of specific traumas or recent events, we examined the impact of stressful events that have accumulated over the lifetime of the youth. Further, as opposed to work in typically developing samples, which tends to define “traumas” by whether specific events as defined in the DSM-5 have occurred, we took a different approach by asking parents to rate how severely youth were affected by each event. For individuals with ASD, it is problematic to decide a priori which major events are “traumatic” and “non-traumatic.” This is because difficulties in adaptive skills, insight, or engagement among these individuals could cause an objectively benign event to be experienced as traumatic, or a traumatic event to be experienced as benign [22, 37]. By using parental ratings of reaction severity to define trauma, we addressed this issue, while providing new information about which stressful life events were most likely to be experienced as traumatic by youth with ASD.

This study also extends the literature by carefully measuring psychopathology using multiple measures and reporters, including a gold-standard clinical interview along with informant- and self-reports of symptomatology. Finally, this study focuses on a time of the lifespan that is of critical importance to youth with ASD and their families yet is highly under-researched—the transition to adulthood. Understanding which youth are likely to develop co-occurring mood or anxiety problems will help to identify those who might struggle most and thus require additional support for transition success [38]. Further, restricting the sample to just those who are still in high school avoids the confound of high school exit, which has been found to predict worsening autism symptoms and behavior problems [39].

We had three specific aims. First, we examined the lifetime occurrence of major and potentially traumatic events among transition-aged youth with ASD, including which events were most often experienced as traumatic. Second, we reported the frequencies of lifetime mood and anxiety symptomatology in this sample. Though similar data have been presented before, this study adds to the existing research by triangulating across measurement methods and reporters to develop robust indicators of mood and anxiety problems, while also taking into account symptomatology that might not meet clinical threshold, but is still impairing (which we label “sub-threshold”). Finally, we examined the relations of cumulative life events and trauma with mood and anxiety symptomatology.

## Methods

### Participants and design

The present study included 36 families of youth with ASD who were part of a larger, longitudinal study. The primary inclusion criteria were that the son or daughter with ASD was exiting high school within the next 12 months and had received an ASD diagnosis from an educational or health professional that was confirmed through a laboratory evaluation. Participants were recruited through local clinics and other autism-related research studies, as well as local support groups, service providers, and autism organizations. ASD diagnoses were confirmed by clinicians with expertise in ASD diagnosis who used a combination of scores from the Autism Diagnostic Observation Schedule [40] administered to the youth and the Autism Diagnostic Interview-Revised [41] administered to the responding parent; all clinicians had achieved external research reliability in these instruments.

The intent of the larger project was to collect data from families of youth across the spectrum of functioning, resulting in data that are more likely to be generalizable to the population of youth in this age range. Because some of these youth would be minimally verbal or have very low IQ scores, parent report was our primary method of data collection. We also collected data directly from the youth for those constructs in which parent report is likely not to suffice—namely, IQ, structured observations of autism symptoms, and internalizing symptomatology (for those youth who could self-report).

Data in this analysis were collected at the first time point, when youth with ASD were in their last year of high school, except for the Schedule of Affective Disorders and Schizophrenia for School Aged Children—Lifetime Version (K-SADS-PL [42]), which was collected at the second time point (within 12 months after high school exit). Given the large variability of functioning among youth with ASD in this sample, the narrow age range allowed us to control for variability due to age. All study procedures were approved by the Vanderbilt University Institutional Review Board.

The youth averaged 18.7 years of age (SD = 1.3), with a range from 17.6 to 22.0 (note that youth with ASD can stay in school until they are 22 years old, although many exit high school with their peers). Over 80 % (83.3 %) were male, and the majority was white non-Hispanic (91.7 %). Most youth had fluent speech (*n* = 31, 86.1 %); 4 were non-verbal and one youth spoke in 2–3 word phrases. IQ scores ranged from 40 to 137; 27.8 % (*n* = 10) had IQs of 70 or less, 11.1 % (*n* = 4) had scores between 71 and 85; 27.8 % (*n* = 10) had scores between 86 and 100, and one third (*n* = 12) had IQ scores that were greater than 100. All youth were living with the responding parent.

The parent sample was composed of 32 mothers and 4 fathers. Parents ranged from 38 to 59 years of age (*M* = 49.2, SD = 4.9). This was a well-educated and well-resourced sample on average; 69 % of the responding parents attained a postsecondary degree (Associate’s or Bachelor’s), and 25 % had earned a post-bachelor’s degree. The median household income was around $80,000, although one quarter of the sample had annual household incomes below $40,000.

### Measures

#### Major and potentially traumatic life events

Using questions derived from Turner and colleagues’ large-scale studies [18, 28, 43], parents were asked whether each of 27 major and potentially traumatic life events had happened to the youth with ASD at any point in his/her lifetime. A full list of the queried events is provided in Table 1. For each event experienced, parents were asked to report how affected the youth was by that event on a scale of 1 (not at all) to 5 (extremely). Any event in which the parent reported that the youth was “extremely” affected (i.e., a rating of “5”) was considered a traumatic event (termed “trauma” or “traumatic experience”); this allowed us to focus on the most clear and significant responses for a more conservative estimate of trauma in this sample. In the present analyses, we calculated a sum of all life events reported by parents (termed “cumulative life events;” possible range from 0 to 27), as well as a binary variable indicating whether the youth had experienced any trauma (1 = youth had at least 1 traumatic experience; 0 = youth had no traumatic experiences). Rationale for treating trauma as a binary variable can be found in Additional File 1.

**Table 1:** Frequency of each life event and the percentage who experienced the event as trauma

Life events	*n* (%) who experienced this	Percentage of those who experience this event who rated it as traumatic
Has anyone else close to youth ever died as a result of a serious accident, injury, or illness?	20 (55.6)	25.0
Has anyone in the home ever had a serious accident, injury, or illness that was life threatening or caused long-term disability?	18 (50.0)	27.8
Did you go through a divorce or separation at any point in youth’s life?	11 (30.6)	27.3
Has anyone in the home ever not had a job for a long time when he/she wanted to be working?	11 (30.6)	9.1
Has youth ever been bullied by his/her peers to such an extent that he/she had to go to the doctor or you considered changing schools?	10 (27.8)	50.0
Has anyone else close to youth ever had a serious accident, injury, or life-threatening illness (but lived)?	10 (27.8)	10.0
Has anyone in the home ever been sent away or kicked out of the house because he/she did something wrong?	6 (16.7)	50.0
Has anyone in the home ever been sexually, physically, or emotionally abused?	5 (13.9)	60.0
Has your family ever been in a major fire, flood, earthquake, or other natural disaster?	5 (13.9)	40.0
Was youth ever forced to live apart from 1 or both parents?	4 (11.1)	100
Has anyone in the home struggled with substance abuse or addiction?	4 (11.1)	50.0
Has anyone in the home ever been physically assaulted or mugged?	4 (11.1)	50.0
Was youth ever abandoned by 1 or both parents?	4 (11.1)	0
Have you ever lost your home because of a natural disaster?	3 (8.3)	66.7
As a child, did youth ever live in an orphanage, foster home, or group home, or was he/she ever a ward of the state?	3 (8.3)	33.3
Has anyone in the home ever been shot at with a gun or threatened with another weapon?	3 (8.3)	33.3
Has youth ever been told that someone else he/she was close to had taken his/her own life?	3 (8.3)	33.3
Has youth ever failed a grade in school?	3 (8.3)	0
Has anyone in the home, or someone youth is close to ever been incarcerated?	3 (8.3)	0
Has youth ever gone through a difficult breakup?	3 (8.3)	0
Has anyone in the home ever taken his/her own life?	2 (5.6)	100
Has youth witnessed anyone in the home being sexually, physically, or emotionally abused?	2 (5.6)	100
Did youth ever discover that a girlfriend or boyfriend was unfaithful?	1 (2.8)	0
Has youth ever been told that someone he/she was close to had been killed?	1 (2.8)	0
Has anyone in the home ever been killed?	0	0
Has anyone in the home ever died as a result of a serious accident, injury, or illness?	0	0
Has youth ever witnessed something violent happen to someone or seen someone killed?	0	0

### Co-occurring mood or anxiety symptomatology

Two variables were constructed to capture whether youth had co-occurring mood symptomatology or co-occurring anxiety symptomatology. For each class of disorder, a number of parent report and self-report measures were used to determine whether the youth had clinical-level, sub-threshold, or no symptomatology.

*Clinical-level anxiety or mood symptomatology* was determined using the informant report version of the K-SADS-PL [42], administered to parents by a trained clinician. The K-SADS-PL is a gold-standard, semi-structured diagnostic interview that assesses current and past episodes of psychopathology in children and adolescents according to DSM-IV criteria. To determine whether an anxiety or mood disorder is present, a screening interview is administered, with follow-up diagnostic supplements. Youth were coded as having *clinical-level mood* symptomatology if they met lifetime (including current) DSM-IV criteria for major depression, dysthymia, and/or bipolar disorder. Youth were coded as having *clinical-level anxiety* symptomatology if they met lifetime (including current) DSM-IV criteria for generalized anxiety disorder, obsessive-compulsive disorder, specific phobia, separation anxiety disorder, panic disorder, and/or post-traumatic stress disorder. We chose to include both lifetime and current criteria so that we would capture youth who may have previously developed psychiatric symptomatology but whose symptoms have remitted or are successfully controlled through psychotropic medications.

We used a number of measures to capture whether youth had *sub-threshold mood* or *sub-threshold anxiety* symptomatology. To be coded in these categories, at least two of the following criteria were met (consistent within type of disorder): (a) report of symptoms on the K-SADS-PL screener that triggered a K-SADS-PL affective or anxiety disorders supplement but without meeting DSM criteria for the corresponding disorder; (b) parent report of a mood or anxiety disorder diagnosed by a medical provider or psychologist, using the psychiatric disorders section of the Rochester Health Status Survey [44], a medical survey designed for individuals with disabilities; (c) youth met clinical cut-offs on the anxiety or depression DSM subscales of the parent-rated Adult Behavior Checklist (ABCL [45]); (d) youth self-reported depressive or anxiety symptomatology that exceeded clinical cut-offs on the Centers for Epidemiological Studies Depression scale (CES-D; scores ≥16 [46]) or the Beck Anxiety Index (BAI; scores ≥16 [47]). Similar to our above rationale for focusing on lifetime symptoms on the K-SADS-PL, we included youth in the *sub-threshold* category if they had received a previous mood or anxiety diagnosis and were currently being treated with psychotropic medication for that disorder (even if their current symptomatology measures were not elevated).

Two youth did not have K-SADS-PL data. In those cases, we decided that neither a previous diagnosis nor current questionnaires on their own were strong enough evidence for the clinical category, and ultimately defined clinical-level symptomatology in these cases as a previous diagnosis plus (a) psychotropic medication specifically taken for that diagnosis, and/or (b) current parent *and* self-report that exceed clinical threshold. Criteria for sub-threshold symptomatology remained the same.

### Data analysis

We used descriptive statistics to examine the distribution of cumulative life events and traumatic experiences (including the frequency of each life event and the proportion of youth who experienced that event as traumatic) as well as the frequencies of mood and anxiety symptomatology (clinical-level, sub-threshold, and none for each type). To examine the relations between cumulative live events, trauma, and mood and anxiety symptomatology, we first ran bivariate statistics. Analysis of variance was used to examine the relations between co-occurring symptomatology and the number of events, and chi-squares were used to examine the relations between co-occurring symptomatology and the presence/absence of trauma. We next used ordinal logistic regression models to test whether significant bivariate relations remained after controlling for the sex of the youth with ASD and his/her IQ (Full-Scale Standardized IQ using the Stanford-Binet Intelligence Scale [48]). We statistically controlled for these variables because females (vs. males) and those with higher (vs. lower) IQ scores in this sample were more likely to have mood disorder symptomatology, *χ*^2^(2) = 7.37, *p* < 0.05 for sex; Spearman rho = 0.44, *p* < 0.01 for IQ.

## Results

### Frequencies of cumulative life events and traumatic experiences

**Fig. 1:**
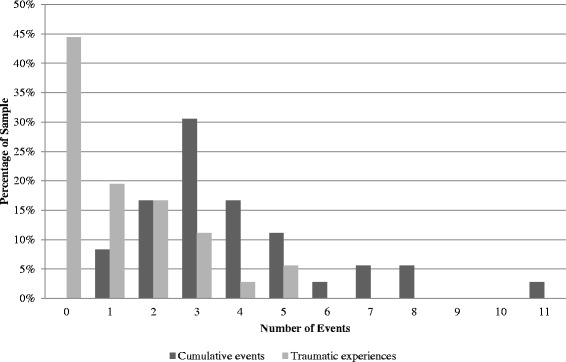
Distribution of cumulative life events and traumatic experiences

Figure 1 presents the distributions of the sum of life events and traumatic experiences. Every youth in the sample experienced at least one life event, with a maximum of 11 and an average around four events per person (*M* = 3.86, SD = 2.19). Twenty-eight percent of youth experienced five or more events. The number of traumatic experiences ranged from 0 to 5, with 55.6 % of youth experiencing at least one trauma.

Table 1 shows the frequency of each type of life event, as well as the conditional percentage of youth who experienced each event as traumatic (i.e., the number who experienced the event as traumatic divided by all youth who experienced the event). The most common events were death of someone close to youth as a result of accident, injury, or illness (56 %); life-threatening injury or illness of someone in the home (50 %); parental divorce/separation (31 %); and unemployment within the home (31 %). There were three events that were reported as traumatic every time they occurred: youth being forced to live apart from one or both parents; death of someone in the home by suicide; and witnessing abuse.

### Frequencies of co-occurring mood and/or anxiety symptomatology

Table 2 presents the frequencies of clinical-level and sub-threshold mood and anxiety symptomatology, as well as which criteria were met in sub-threshold cases. The most common mood disorder was major depression, and the most common anxiety disorder was obsessive-compulsive disorder. None of the youth met the criteria for post-traumatic stress disorder (PTSD). Of those youth meeting clinical criteria who were able to self-report (*n* = 15), two thirds either had elevated symptoms on the corresponding self-report measure (CES-D or BAI; 53.3 %, *n* = 8) or were taking psychotropic medications targeting symptoms of the corresponding psychiatric disorder (but did not currently have elevated self-reported symptoms; 13.3 %, *n* = 2). Of those youth who met sub-threshold criteria, the vast majority had a previous mood or anxiety disorder diagnosis from a medical professional in addition to elevated symptom levels on questionnaires (see Table 2). About 40 % of youth in the sub-threshold categories met criteria by *both* parent and self-report.

**Table 2:** Descriptive information on co-occurring mood and anxiety symptomatology

Mood	Anxiety
Clinical level (25.0 %, *n* = 9)	Clinical level (25.0 %, *n* = 9)
- 5 major depression	- 4 obsessive-compulsive disorder
- 2 dysthymia	- 3 simple phobia
- 1 bipolar disorder	- 2 generalized anxiety disorder
- 1 bipolar disorder + depressive disorder NOS	
Sub-threshold level (13.8 %, *n* = 5)	Sub-threshold level (22.2 %, *n* = 8)
- 2 previous diagnosis + K-SADS-PL supplement	- 3 K-SADS- PL supplement + exceeded BAI cut-off
- 1 previous diagnosis + exceeded CES-D cut-off	- 2 previous diagnosis + K-SADS-PL supplement
- 1 previous diagnosis + exceeded ABCL depression cut-off + exceeded CES-D cut-off	- 2 previous diagnosis + exceeded ABCL anxiety cut-off
- 1 K-SADS-PL supplement + exceeded ABCL depression cut-off	- 1 previous diagnosis + psychotropic meds (anti-anxiety)
None (61.1 %, *n* = 22)	None (52.7 %, *n* = 19)

*CES-D* Centers for Epidemiological Studies Depression scale, *K-SADS-PL* Schedule of Affective Disorders and Schizophrenia for School Aged Children–Lifetime Version, *BAI* Beck Anxiety Index, *ABCL* Adult Behavior Checklist

Table 3 presents the overlap between mood and anxiety symptomatology. Nearly 50 % (47.3 %) of youth met DSM criteria for a lifetime mood and/or anxiety disorder (i.e., clinical-level symptomatology); only one participant met criteria for both in this sample. One third of youth (*n* = 12) had no evidence of mood or anxiety disorders, meaning that they did not meet clinical or sub-threshold criteria on either.

**Table 3:** Co-occurrence of lifetime mood and anxiety symptomatology

	Anxiety symptomatology			
Mood symptomatology	None	Sub-threshold	Clinical	Total
None	*n* = 12	*n* = 4	*n* = 6	*n* = 22
	33.3 %	11.1 %	16.7 %	61.1 %
Sub-threshold	*n* = 2	*n* = 1	*n* = 2	*n* = 5
	5.6 %	2.8 %	5.6 %	13.9 %
Clinical	*n* = 5	*n* = 3	*n* = 1	*n* = 9
	13.9 %	8.3 %	2.8 %	25.0 %
Total	*n* = 19	*n* = 8	*n* = 9	*n* = 36
	52.8 %	22.2 %	25.0 %	100 %

### Relations between cumulative life events, trauma, and mood/anxiety symptomatology

Findings from bivariate analyses of cumulative life events, presence/absence of trauma, and mood and anxiety symptomatology are presented in Table 4. The relations between co-occurring symptomatology (mood and anxiety) and number of events were not statistically significant; however, having at least one trauma was related to a greater likelihood of mood problems. Of those youth with clinical-level mood symptomatology, nearly 90 % had experienced at least one trauma (compared to about 40 % of those with no symptomatology). After taking IQ and sex into account, the presence of trauma remained related to greater mood symptomology, *B* = 1.88, OR = 6.57 (95 % CI = 0.99–43.55), Wald *χ*^2^ = 3.81, *p* = 0.051. These data suggest that, although a significant proportion of youth with trauma do not have a co-occurring mood disorder, these disorders were rarely observed in the absence of an event that is experienced as traumatic.

**Table 4:** Bivariate relations between cumulative life events, trauma, and co-occurring mood or anxiety symptomatology

	Mean (SD) or % for clinical-level symptoms	Mean (SD) or % for sub-threshold level symptoms	Mean (SD) or % for no symptoms	*F* value/χ^2^
Mood				
Cumulative events	4.67 (2.87)	3.00 (1.87)	3.72 (1.93)	1.04
Percent with a trauma	88.9 %	60.0 %	40.9 %	6.00*
Anxiety				
Cumulative events	3.00 (1.66)	5.38 (2.97)	3.63 (1.80)	3.02^†^
Percent with a trauma	33.3 %	75.0 %	57.9 %	3.07

^†^ *p* ≤ 0.10; *p ≤ 0.05

### Follow-up analyses

Although our data is cross-sectional, our hypothesized direction of effects was that experiencing a trauma would lead to mood or anxiety symptomatology. To explore the issue of timing, we examined the age of onset for those with clinical-level mood or anxiety symptomatology and compared that to the age of the first life event. Note that we consider these analyses exploratory because they are based on retrospective reports. Clinical-level anxiety disorders emerged, on average, around 5 years of age compared to around 13 years of age for mood disorders. For those with a mood disorder, in every case, the first life event happened before or at the same time as the onset of the disorder. However, there were many cases in which the onset of an anxiety disorder preceded the first life event—particularly for those who met criteria for specific phobias or obsessive-compulsive disorder.

## Discussion

Our findings suggest that mood symptomatology in transition-aged youth with ASD is often related to an event (or events) experienced as traumatic at some point in childhood/ adolescence. The rates of trauma that we observed when stratifying by mood symptomatology (88.9 % of those with clinical-level symptomatology had a traumatic experience, compared to 40.9 % of those with no mood symptomology) were nearly identical to those found by Ghazuiddin and colleagues [31]—the only other study to our knowledge to look at the mental health implications of cumulative stressful life events for individuals with ASD. Trauma or cumulative life events were not, however, related to anxiety symptomatology in our sample. Compared to mood symptoms within the autism spectrum, significant anxiety is more likely to develop earlier in childhood, which blurs the association with later-occurring life events. In that case, it is possible that biological or other early developmental mechanisms may be weighted more heavily than purely psychosocial ones in the development of anxiety within ASD, particularly for the types most common in this sample (obsessive-compulsive disorder and simple phobia, which share features with the autism phenotype itself). The development of major depression, on the other hand, may be more experience-dependent than anxiety [49]. Given reportedly high rates of mood disorder in first-degree relatives of individuals with ASD, offspring with ASD may have a similar predisposition to mood problems which is then “kindled” by stressful life events.

Although youth in our sample without a traumatic experience rarely met criteria for lifetime clinical-level mood symptomatology, there were many cases in which youth had a trauma but did not develop psychiatric comorbidities. This suggests that some youth with ASD may be more resilient to the effects of trauma. The pathway by which traumatic experiences influence psychopathology may be shaped by emotion regulation problems, which are common to ASD [50]. Emotion regulation difficulties can be secondary characteristics, such as poor coping skills, or more autism-specific. For example, Gotham and colleagues [13] found that adults with ASD who perceived greater impact of ASD symptoms on their lives tended to have elevated depressive symptoms if they also reported higher levels of cognitive perseveration (rumination). Similarly, major life events might be more commonly experienced as traumatic in those individuals who are predisposed to perseverate on such events, and this perseveration may in turn contribute to a mood disorder for some with ASD. Although rumination was not measured in this study, future research should examine how it might influence the impact of traumatic experiences for youth with ASD.

In addition to individual factors, family factors likely play an important role in promoting resiliency for youth with ASD who experience traumatic events. Studies suggest, for example, that individuals with greater social support and economic resources more often experience resilience in the face of trauma [51]. Among children who experience the loss of a parent, warmth in the home is a major predictor of whether they develop psychopathology [52]. Thus, investigation into the factors that promote resiliency to trauma among youth with ASD should consider family characteristics in addition to personal resources.

Our appraisal data provided us with a different view of how youth were affected by major events than what we might have expected had we pre-determined which events would be considered “trauma.” For example, one might consider losing one’s home in a natural disaster or fire as traumatic; but in this sample, it was not always experienced as such (as reported by parents). Recent reviews on trauma among individuals with disabilities [22, 37] suggest that the individuals’ appraisals of the event may be more important than the occurrence or accumulation of events. Our findings suggest that this is indeed the case: it was the appraisals of events that were related to mood symptomatology and not the accumulation of events. Thus, studies focused on trauma in ASD should include measures of the impact of events.

The inclusion of multiple measures of mood and anxiety symptomatology in our study points to the dramatic differences in rates of mood/anxiety problems depending on what measure is being used (further information about the correspondence between measures are presented in supplemental material). Our estimates of clinical-level symptomatology, using the K-SADS-PL, were lower than some other studies of adolescence/adults with ASD (see introduction). This might be because our sample was recruited from the community and thus truly has lower rates of comorbidities than clinic samples [5]. However, when we added sub-threshold symptomatology, the rates were much higher. Notably, any instrument or indicator used singularly yielded different interpretations of comorbidity rates. For example, 25 % of our sample met DSM lifetime criteria for an anxiety disorder on the K-SADS-PL, one third had received an anxiety disorder from a medical professional, 23 % had moderate or severe self-reported anxiety symptoms on the BAI, and 6 % had clinical-level parent-reported anxiety symptoms on the ABCL. Given the very different rates of psychiatric problems in one sample based on the measure being used, comparing rates of psychiatric disorders among different samples using different instruments is problematic. In order to best understand rates of comorbidities in ASD, multiple instruments with multiple reporters will likely be necessary [53].

This study has a number of limitations that are offset by important strengths. First, as in much of ASD research, our measure of major and potentially traumatic life events was collected via parent report. Relying on informants runs the risk of missing life events that parents do not know about or differing appraisals of what constitutes a “trauma” between parent and son/daughter. One next step in this line of research would be to explore the correspondence in reports of trauma across parents and youth with ASD. One could also observe the impact of events as they emerge to determine which reporter seems to be the most reliable. As many of our measures of psychiatric symptomatology were also informant report, relationships between our variables might be inflated by informant bias. Despite these drawbacks, the reliance on informant report was necessary because we included youth with ASD across the spectrum of functioning (some of whom are non-verbal, had very low IQ scores, or otherwise could not self-report).

Though we attempted to make best-estimate standardized diagnoses of psychiatric comorbidity by choosing a gold-standard instrument (the K-SADS-PL), an autism-specific instrument, such as the Autism Comorbidity Interview [54], may have yielded more accurate results, particularly for participants with ID. We attempted to improve the validity of our comorbid diagnoses by using a multi-method, multi-rater approach. Ultimately, a significant strength of this study is the thoroughness by which we measured co-occurring mood and anxiety symptomatology, including a structured clinical interview, parent report of symptoms and previous diagnoses, youth self-report, and medication use.

The small sample in this study is another limitation, as it raises concerns about the generalizability of our findings. On the other hand, because this was part of a larger longitudinal project, and advertised only for the high school exit criteria (youth had to be in their last year of high school), the results may be more broadly indicative of mental health issues in this age range, rather than being subject to self-selection bias of studies of emotional health and/or trauma in ASD. Furthermore, this sample, though small, was extremely well-characterized using gold-standard diagnostic and IQ measures. Nevertheless, there may be a relationship between trauma and anxiety symptomatology in ASD that we could not detect due to sample size. In particular, this study likely lacked sufficient power to explore comparatively less common mental health outcomes (e.g., PTSD, bipolar disorder, schizophrenia) in this special population.

The wide range of functioning of the youth with ASD included in this sample also aids generalizability. It should be noted that it can be more difficult to detect mood disorders among minimally verbal adults [55], and it also might be more difficult for parents of these individuals to discern the impact of potentially traumatic events; however, in this particular sample, there were no statistically significant differences in clinical mood symptomatology or the presence of trauma based on whether the youth had ID. Finally, because this was a retrospective study, we were dependent on parents’ recollection of the timing of major life events and onset of psychiatric symptomatology. Although our follow-up analyses suggested that the onset of the first major life event likely preceded the onset of mood symptomatology, only a longitudinal study can truly examine how these processes unfold over time.

## Conclusions

Our findings suggest that contextual factors such as trauma might be important for the development of psychopathology (specifically mood symptomatology) in individuals with ASD. Although this idea is well-accepted in typically developing populations, contextual factors are rarely studied in investigations of psychopathology or transition outcomes in ASD. Given the high rates of psychiatric comorbidities in this population, future research should continue to identify the range of possible factors—both behavioral and contextual—that might influence the emergence of these disorders. This can help us to identify those youth who are most likely to develop comorbidities and thus struggle during transition, as well as help to create and refine novel treatment options.

## Abbreviations

ABCL, Adult Behavior Checklist; ASD, autism spectrum disorder; BAI, Beck Anxiety Index; CES-D, Centers for Epidemiological Studies Depression scale; ID, intellectual disability; K-SADS-PL, Schedule of Affective Disorders and Schizophrenia for School Aged Children–Lifetime Version

## Additional file

Additional file 1: Supplemental Material. (DOCX 31.9 kb)
