# Supplementary material for: Cumulative life events, traumatic experiences, and psychiatric symptomatology in transition-aged youth with autism spectrum disorder
Source: J Neurodev Disord. 2016 Jul 27;8:28. doi: 10.1186/s11689-016-9160-y (PMC4962443; doi:10.1186/s11689-016-9160-y)
Supplement: Additional file 1: — Supplemental Material. (DOCX 31.9 kb) [file 11689_2016_9160_MOESM1_ESM.docx]

**Supplemental Material**

**Treating the number of traumas as continuous vs. a binary variable**

In initial analyses, we explored whether our measure of traumatic events should be treated as a continuous variable (i.e., the number of traumas) versus a binary variable indicating whether the youth had experienced at least one trauma. To do this, we ran a chi-square analyses to determine whether each additional trauma seemed to confer additional risk for mood disorders (this was less relevant for anxiety disorders, as there was no relationship between trauma and anxiety disorders). As can be seen from Figure 1, fewer than 10% of participants had more than 3 traumatic events; thus, we truncated our continuous measure of trauma at 3 or more. Of those youth who had no traumatic events, 6.3% met criteria for a clinical mood disorder, compared to 42.9% of those with 1 traumatic event, 33.3% of those with 2 traumatic events, and 42.9% of those with 3 or more traumatic events. Thus, it appeared that the experience of any trauma was related to mood problems, and multiple traumas did not seem to increase the likelihood of association in this sample. Therefore, in our main analyses we chose to analyze trauma as a binary variable (0 = no traumatic experiences; 1 = at least one traumatic experience).

**Examining correspondence between mood and anxiety measures**

The percentage of youth who met cut-offs for mood or anxiety symptoms on each instrument are presented in Table S1. The parent-reported ABCL cut-off was a more conservative indicator of emotional health problems compared to self-report, particularly for mood (CES-D). Parent-reported history of a professional diagnosis and positive status for taking psychotropic medications was endorsed at similar rates, at approximately one-third of the sample. Additionally, about one-third or more participants screened into the relevant KSADS supplement (mood or anxiety), and 25% of the total sample (comprising the majority of those who screened into the supplement) received the relevant diagnosis on that standardized interview.

Tables S2, S3, and S4 examined correspondence between indicators of anxiety (S2, S3) and mood (S2, S4) problems. For both anxiety and mood problems, current self-report and “ever” (including current) professional diagnosis were less likely to agree (around 50-60%) than other combinations of indicators (see Table S2). Where they disagreed, current self-report was more likely than ever-diagnosis to indicate mood symptoms, and ever-diagnosis was more likely than self-report to be positive for anxiety (see Tables S3 and S4). This is probably influenced by the developmental time course of both disorders, in that depression is more likely than anxiety to have adolescent or adult onset. Nevertheless, it is possible that people with ASD are more likely to receive a comorbid anxiety diagnosis from a professional, whereas depression may be overlooked by comparison.

In general, when indicators of emotional health problems disagree (see Tables S3 and S4), we usually observed a relatively even split in which both instruments indicated problems in different cases, with the exception of the more conservative ABCL, which was substantially more likely to be negative (i.e., participant did not meet cut-off for clinical concern) than most other indicators for both anxiety and mood.

For anxiety problems (Tables S2 and S3), medication status tended to not agree highly with screening into the KSADS supplement or exceeding thresholds on the BAI, perhaps because psychotropic medication use is not specific to anxiety or because the time frame of problems versus treatment and remission was neither clear nor standardized across participants. In contrast to the parent-reported ABCL findings described above, the clinician-rated parent-report interview (KSADS) was more likely to indicate anxiety problems than was self-report (BAI).

Indicators of mood problems (see Tables S2 and S4) evidenced slightly higher agreement than indicators of anxiety. Self-report (CES-D) was more commonly positive for mood problems over parent report (meeting ABCL or KSADS diagnosis criteria).

Supplemental Table 1

Percentage of youth for whom mood or anxiety problems were indicated by measure

|  | Mood | Anxiety |
| --- | --- | --- |
| 1. Parent-reported history of mood or anxiety diagnosis (possible n=36) | 30.6%  (n=11) | 33.3%  (n=12) |
| 1. Received KSADS mood or anxiety disorder supplement (possible=34) | 32.4%  (n=11) | 44.1%  (n=15) |
| 1. Met clinical-cut off parent-reported mood or anxiety symptoms on ABCL (possible n=36) | 13.9%  (n=5) | 5.6%  (n=2) |
| 1. Self-reported mood symptoms above cut-off on the CES-D (possible n=29) or anxiety symptoms in the moderate/severe range on the BAI (possible n=30) | 41.4%  (n=12) | 23.3%  (n=7) |
| 1. Current taking anti-anxiety or anti-depressant medication (possible n =36) | 33.3%  (n=12) | |
| 1. Met criteria on KSADS for a mood or anxiety disorder (possible n=34) | 23.5%  (n=8) | 26.5%  (n=9) |

*Note*. KSADS = Schedule of Affective Disorders and Schizophrenia for School Aged Children – Lifetime Version; ABCL = Adult Behavior Checklist; BAI = Beck Anxiety Inventory; CES-D = Centers for Epidemiological Studies Depression Scale

Supplemental Table 2

Percent agreement (both yes or both no) between anxiety (above diagonal) and mood (below diagonal) measures

|  | 1. Diagnosis | 2. KSADS supplement | 3. ABCL | 4. Self-report | 5. Medications | 6. KSADS Diagnosis |
| --- | --- | --- | --- | --- | --- | --- |
| 1. Parent-reported history of diagnosis | ---------- | 58.8% | 72.2% | 56.7% | 66.7% | 64.7% |
| 1. Received KSADS supplement | 79.4% | ---------- | 52.9% | 71.4% | 41.2% | ^a.^ |
| 1. Met clinical-cut off on ABCL | 72.2% | 73.5% | ---------- | 70.0% | 72.2% | 70.6% |
| 1. Elevated self-reported symptoms | 58.6% | 63.0% | 62.1% | ---------- | 53.3% | 67.9% |
| 1. Current taking anti-anxiety or anti-depressant medication | 63.9% | 64.7% | 80.6% | 55.2% | ---------- | 52.9% |
| 1. Met criteria on KSADS for lifetime diagnosis | 76.5% | ^a.^ | 76.5% | 74.1% | 67.6% | ---------- |

^a.^ Instead of reporting correspondence for KSADS supplement vs. KSADS diagnosis, we reported the percentage of youth with symptoms severe enough to warrant the disorder supplement (either mood or anxiety) who also met clinical criteria for a mood or anxiety disorder on this instrument. Of 15 youth who received the anxiety disorders supplement, 9 met KSADS criteria for an anxiety disorder (60%). Of 11 youth who received the mood disorders supplement, 8 met KSADS criteria for a mood disorder (72.7%).

*Note*. Two families did not complete the KSADS, and some youth were unable to complete the self-report measures, resulting in different denominators. KSADS = Schedule of Affective Disorders and Schizophrenia for School Aged Children – Lifetime Version; ABCL = Adult Behavior Checklist.

Supplemental Table 3

Detailed information on agreements (above diagonal) and disagreements (below diagonal) in the anxiety measures

|  | 1. DX | 2. KSADS-SUP | 3. ABCL | 4. BAI | 5. MEDS | 6. KSADS-DX |
| --- | --- | --- | --- | --- | --- | --- |
| 1. Parent-reported history of anxiety diagnosis (DX) | ---------- | 18% agree yes  41% agree no | 6% agree yes  67% agree no | 7% agree yes  50% agree no | 17% agree yes  50% agree no | 12% agree yes  53% agree no |
| 1. Received KSADS anxiety disorder supplement   (KSADS-SUP) | 15%  - DX yes  - KSADS-SUP no 27%  - DX no  - KSADS-SUP yes | ---------- | 0% agree yes  53% agree no | 21% agree yes  50% agree no | 9% agree yes  32.4% no | 27% agree yes  56% agree no |
| 1. Met clinical-cut off for anxiety problems on ABCL (ABCL) | 28%  - DX yes  - ABCL no  0%  - ABCL no  - DX yes | 44%  - KSADS-SUP yes  - ABCL no  3%  - KSADS-SUP no  - ABCL yes | ---------- | 0% agree yes  70% agree no | 6% agree yes 67% agree no | 0% agree yes 71% agree no |
| 1. Elevated self-reported symptoms on BAI (BAI) | 27%  - DX yes  - BAI no  17%  - DX no  - BAI yes | 29%  - KSADS-SUP yes - BAI no  0%  - KSADS-SUP no - BAI yes | 7%  - ABCL yes  - BAI no  23.3%  - ABCL no  - BAI yes | ---------- | 3% agree yes 50% agree no | 11% agree yes 57% agree no |
| 1. Current taking anti-anxiety or anti-depressant medication (MEDS) | 17%  - DX yes  - MEDS no  17%  - DX no  - MEDS yes | 35%  - KSADS-SUP yes - MEDS no  24%  - KSADS-SUP no - MEDS yes | 0%  - ABCL yes,  - MEDS no  28%  - ABCL no  - MEDS yes | 20%  - BAI yes  - MEDS no  27%  - BAI no  - MEDS yes | ---------- | 6% agree yes 47% agree no |
| 1. Met criteria on KSADS for lifetime anxiety disorder diagnosis   (KSADS-DX) | 21%  - DX yes  - KSADS-dx no  15%  - DX no  - KSADS-DX yes | 18%  - KSADS-SUP yes - KSADS-DX no  (must have supplement for diagnosis) | 3%  - ABCL yes  - KSADS-DX no  27%  - ABCL no  - KSADS-DX yes | 11%  - BAI yes  - KSADS-DX no  21%  - BAI no  - KSADS-DX yes | 27%  - MEDS yes  - KSADS-DX no  21%  - MEDS no  - KSADS-DX yes | ---------- |

*Note*. KSADS = Schedule of Affective Disorders and Schizophrenia for School Aged Children – Lifetime Version; ABCL = Adult Behavior Checklist; BAI = Beck Anxiety Inventory. The four percentages in corresponding cells across the diagonal total to 100% of participants who received both questions/instruments (i.e., Agree Yes + Agree No + Yes1,No2 + Yes2,No1).

Supplemental Table 4

Detailed information on agreements (above diagonal) and disagreements (below diagonal) in the mood measures

|  | 1. DX | 2. KSADS-SUP | 3. ABCL | 4. CESD | 5. MEDS | 6. KSADS-DX |
| --- | --- | --- | --- | --- | --- | --- |
| 1. Parent-reported history of mood diagnosis (DX) | ---------- | 21% agree yes  59% agree no | 8% agree yes  64% agree no | 17% agree yes  41% agree no | 14% agree yes  50% agree no | 15% agree yes  62% agree no |
| 1. Received KSADS mood disorder supplement (KSADS-SUP) | 9%  - DX yes  - KSADS-SUP no  12%  - DX no  - KSADS-SUP yes | ---------- | 9% agree yes  65% agree no | 19% agree yes 44% agree no | 15% agree yes 50% agree no | 24% agree yes 68% agree no |
| 1. Met clinical-cut off for depressive problems on ABCL (ABCL) | 22%  - DX yes  - ABCL no  6%  - DX no  - ABCL yes | 24%  - KSADS-SUP yes - ABCL no  3%  - KSADS-SUP no - ABCL yes | ---------- | 10% agree yes  52% agree no | 14% agree yes 67% agree no | 6% agree yes  71% agree no |
| 1. Elevated self-reported symptoms on CES-D (CES-D) | 17%  - DX yes  - CESD no  24%  - DX no  - CESD yes | 15%  - KSADS-SUP yes - CES-D no  22%  - KSADS-SUP no - CES-D yes | 7%  - ABCL yes  - CES-D no  31%  - ABCL no - CES-D yes | ---------- | 14% agree yes 41% agree no | 19% agree yes 56% agree no |
| 1. Current taking anti-anxiety or anti-depressant medication (MEDS) | 17%  - DX yes  - MEDS no  19%  - DX no  - MEDS yes | 18%  - KSADS-SUP yes - MEDS no  18%  - KSADS-SUP no  - MEDS yes | 0%  - ABCL yes - MEDS no  19%  - ABCL no - MEDS yes | 28%  - CES-D yes - MEDS no  17%  - CES-D no - MEDS yes | ---------- | 12% agree yes 56% agree no |
| 1. Met criteria on KSADS for lifetime mood disorder diagnosis (KSADS-DX) | 15%  - DX yes  - KSADS-DX no  9%  - DX no  - KSADS-DX yes | 9%  - KSADS-SUP yes - KSADS-DX no  (must have supplement for diagnosis) | 6%  - ABCL yes - KSADS-DX no  18%  - ABCL no - KSADS-DX yes | 22%  - CES-D yes - KSADS-DX no  4%  - CES-D no - KSADS-DX yes | 21%  - MEDS yes - KSADS-DX no  12%  - MEDS no - KSADS-DX yes | ---------- |

*Note*. KSADS = Schedule of Affective Disorders and Schizophrenia for School Aged Children – Lifetime Version; ABCL = Adult Behavior Checklist; CES-D = Centers for Epidemiological Studies Depression Scale. The four percentages in corresponding cells across the diagonal total to 100% of participants who received both questions/instruments (i.e., Agree Yes + Agree No + Yes1,No2 + Yes2,No1).
